# Effects of Pyramid Resistance-Training System with Different Repetition Zones on Cardiovascular Risk Factors in Older Women: A Randomized Controlled Trial

**DOI:** 10.3390/ijerph17176115

**Published:** 2020-08-22

**Authors:** Leandro dos Santos, Alex S. Ribeiro, João Pedro Nunes, Crisieli M. Tomeleri, Hellen C. G. Nabuco, Matheus A. Nascimento, Paulo Sugihara Junior, Rodrigo R. Fernandes, Francesco Campa, Stefania Toselli, Danielle Venturini, Décio S. Barbosa, Luís B. Sardinha, Edilson S. Cyrino

**Affiliations:** 1 Metabolism, Nutrition, and Exercise Laboratory, Physical Education and Sports Center, Londrina State University, 86057-970 Londrina, PR, Brazil; leandro.santos.sm@gmail.com (L.d.S.); alex.sribeiro@kroton.com.br (A.S.R.); crisielitomeleri@gmail.com (C.M.T.); hellencgarcez@gmail.com (H.C.G.N.); matheusamarante@gmail.com (M.A.N.); juniornutricao@gmail.com (P.S.J.); rodrigo.r.fernandes@gmail.com (R.R.F.); edilsoncyrino@gmail.com (E.S.C.); 2 Center for Research in Health Sciences, University of Northern Paraná, 86041-140 Londrina, PR, Brazil; 3 Paraná State University—UNESPAR, Paranavaí Campus, 87703-000 Paranavaí, PR, Brazil; 4 Department for Life Quality Studies, University of Bologna, 47921 Rimini, Italy; francesco.campa3@unibo.it; 5 Department of Biomedical and Neuromotor Science, University of Bologna, 40126 Bologna, Italy; stefania.toselli@unibo.it; 6 Clinical Analyses Laboratory, Londrina State University, 86057-970 Londrina, PR, Brazil; danielle.venturini@bol.com.br (D.V.); sabatini@uel.br (D.S.B.); 7 Exercise and Health Laboratory, CIPER, Faculdade de Motricidade Humana, Universidade de Lisboa, 1499-002 Lisboa, Portugal; lbsardinha55@gmail.com

**Keywords:** body composition, strength training, cardiometabolic risk, elderly

## Abstract

This study analyzed the effects of the pyramidal resistance training (RT) system with two repetition zones on cardiovascular risk factors in older women (≥60 years old). Fifty-nine older women were randomly assigned in three groups: non-exercise control (CON, *n* = 19), narrow-pyramid system (NPR, *n* = 20), and wide-pyramid system (WPR, *n* = 20). Training was performed for eight weeks (eight exercises for the whole-body, 3x/week) in which NPR and WPR performed three sets of 12/10/8 and 15/10/5 repetitions, respectively. Regional body fat was estimated by dual-energy X-ray absorptiometry, and blood parameters related to glycemic, lipid, and inflammatory profiles were assessed. After the training period, although no difference was observed for the magnitude of the changes between NPR and WPR, significant group by time interactions indicated benefits with RT compared to CON for reducing body fat (mainly android body fat; −7%) and improving glucose, HDL-C, LDL-C and C-reactive protein (*p* < 0.05). Composite z-score of cardiovascular risk, created by the average of the intervention effects on the outcomes, indicate similar responses between NPR and WPR, differing from CON (*p* < 0.001). Results indicate that both the repetition zones of the pyramidal RT reduced similarly the cardiovascular risk in older women.

## 1. Introduction

Cardiovascular diseases (CVD) are the main causes of morbimortality in women worldwide [1]. Menopause and aging are related to increases in low-density lipoprotein cholesterol (LDL-C), ectopic fat accumulation, and body fat redistribution extending from the limb region to the trunk [2,3,4]. Increases in visceral adiposity have been associated with inflammation [5] and recurrent cardiovascular events [4]. This scenario can be counteracted with exercise, whereby resistance training (RT) is recommended as one of the main modalities for older people [6,7,8,9]. Increments in skeletal muscle function and mass are the main RT-related benefits [6], which are related to reduced adiposity levels, by improving resting metabolic rate, energy expenditure, and metabolic activity of muscle tissue [6,7,9].

The effectiveness of RT is associated with the appropriated manipulation of variables related to intensity and volume [6,10,11]. Current evidence suggests that increases in strength are more dependent on the intensity of load, while muscle hypertrophy is related to the RT volume [12,13,14,15,16]. However, the best approach to improve CVD risk factors in older women is still unknown [6,7,17,18]. Recently, our group showed that traditional RT, even at low volume (1 set per exercise, 10–15 repetitions-maximum (RM), 3x/week–non-consecutive days) improved total cholesterol (TC), low-density lipoprotein cholesterol (LDL-C), glucose (GLU), and C-reactive protein (CRP) in untrained older women [19]. Moreover, we observed that performing a higher volume (3 sets per exercise, 10–15 RM) resulted in greater improvements [18].

In another study, we compared the effects of the traditional (three sets of 8–12 RM) vs. the pyramidal (12/10/8 RM in the first, second, and third set, respectively) RT systems for eight weeks on CVD risk factors and observed adaptations of similar magnitudes between them [20]. The crescent-pyramid RT system is a training strategy that allows the combination of high mechanical and metabolic stimuli by combining simultaneous decreases in the volume of repetitions and increases in the intensity of load throughout the sets [20,21,22,23]. However, the progression of the training loads depends on the number of repetitions to be performed. The use of a wider zone (i.e., 15/10/5 RM throughout the three sets) is posited to allow an overload relatively more accentuated then promote better results compared to a narrow repetition-zone (i.e., 12/10/8 RM), as we already tested [20,21]. That is, due to its inherent characteristic of larger variations in loads and the number of repetitions, this approach allows exercise performance at higher overload with similar volume, thus providing a favorable anabolic environment for increasing strength and muscle hypertrophy [22]. Indeed, we have recently observed a benefit for the wide-pyramid compared to the narrow pyramid (15/10/5 RM vs. 12/10/8 RM) for improving muscular strength (e.g., elbow flexors strength = wide: +18%; narrow: +11%) and appendicular muscle mass (wide: +8%; narrow: +4%) [22]. Nonetheless, its effects on other body composition outcomes or CVD risk factors still require further investigation.

Therefore, the purpose of the present study was to compare the effects of the pyramid systems with narrow and wide repetitions zones on CVD risk factors in older women. Considering that higher training volume may induce more significant fat loss [18,24], greater muscle growth may be related to a reduction in CVD risk factors [6,7,9], and the preliminary results on muscle function show a favorable benefit for the wide repetition-zone pyramid [22,23], we hypothesized that the broader repetition-zone pyramid training would induce better adaptations on body fat and blood markers related to glycemic, lipid, and inflammatory profiles.

## 2. Materials and Methods

### 2.1. Experimental Design

The present study is part of a longitudinal research project named “Active Aging Longitudinal Study”, initiated in 2012. Its purpose is to analyze the effects of supervised, structured, and progressive RT programs on neuromuscular, morphological, physiological, and metabolic outcomes in older women [22,23]. A randomized controlled trial was carried out over 12 weeks, with eight weeks dedicated to the RT program, and four weeks for data collection. Pre- and post-intervention testing was carried out at weeks 1–2 and 11–12, respectively, and comprised anthropometric, body composition, and metabolic biomarkers measurements. The RT program was carried out during weeks 3–10. Adherence to the RT program was established as >85% of the total sessions. The procedures were conducted according to the Declaration of Helsinki, and the Londrina State University Ethics Committee approved this investigation (committee opinion number: 1.306.507). No adverse event occurred during the intervention period.

### 2.2. Subjects

Recruitment was carried out through the newspaper, television programs, radio advertisings, and home delivery of leaflets in the central area and residential neighborhoods. Interested individuals completed detailed health history and physical activity questionnaires. Participants were subsequently admitted to the study if they met specific inclusion criteria: female, ≥60 years old, physically independent, free from cardiac dysfunction, not receiving hormonal replacement therapy, and not performing any regular physical exercise for more than once a week over the six months preceding the beginning of the study. Participants passed a diagnostic test by a cardiologist (resting 12-lead electrocardiogram test, personal interview, and treadmill stress test when deemed necessary). All were released with no restrictions for participation in this study. Fifty-nine physically independent older women (67.3 ± 4.4 years, 66.5 ± 12.6 kg, 1.55 ± 0.1 m, 27.6 ± 5.0 kg.m^−^^2^) were selected and randomly assigned to one of three groups: control group (CON, *n* = 19), instructed not to engage in any physical exercise training program during the period of the intervention and to maintain their customary eating and physical activity patterns; pyramid RT system with narrow repetition zone (NPR, *n* = 20), in which participants performed three sets of 12/10/8 RM per exercise, respectively; or pyramid RT system with wider repetition zone (WPR, *n* = 20), in which participants performed three sets of 15/10/5 RM per exercise, respectively. This final number of subjects reached the necessary for this experiment, according to the sample size calculation (repeated measures, moderate effect size = 0.50, α = 0.05, power = 0.80). Written informed consent was obtained from all participants after a detailed description of study procedures was provided.

### 2.3. Body Composition

Total body fat (TBF), android body fat (ABF), and gynoid body fat (GBF) were assessed using a dual-energy X-ray absorptiometry (DXA) scan (Lunar Prodigy, model NRL 41990, GE Lunar, Madison, WI, USA). Before scanning, participants were instructed to remove all objects containing metal. Calibration and scans were performed per the manufacturer’s instruction manual. A laboratory technician carried out both calibration and analysis. Analyses during the intervention were performed by the same technician who was blinded to the intervention. The intraclass correlation coefficient (ICC) for TBF, ABF, and GBF were ≥0.98, while standard error of measurement (SEM) were 0.90 kg, 0.25 kg, and 0.41 kg for TBF, ABF, and GBF, respectively.

### 2.4. Biochemical Analysis

After a 12-h fast, a laboratory technician collected blood samples from each subject from the antecubital vein. The subjects were instructed to avoid alcohol or caffeinated beverages 72 h before collection and not perform the vigorous exercise for the preceding 24 h. Samples were deposited in vacuum tubes with a gel separator without anticoagulant and were centrifuged for 10 min at 3000 rpm for serum separation. Measurements of serum levels of glucose (GLU), total cholesterol (TC), high-density lipoprotein (HDL-C), triglycerides (TG), and high-sensitivity C-reactive protein (CRP) were determined by standard methods in a specialized laboratory at University Hospital. The low-density lipoprotein (LDL-C) was estimated using the Friedewald equation [25]. The analyses were carried out using a biochemical auto-analyzer system (Dimension RxL Max—Siemens Dade Behring) according to established methods in the literature, consistent with the manufacturer’s recommendations.

### 2.5. Resistance Training Program

The supervised RT program was performed three times a week (Mondays, Wednesdays, and Fridays) for over eight weeks. Training took place in the morning and was based on recommendations for RT in an older population to improve muscle hypertrophy and muscular strength [6]. Physical Education professionals personally supervised (1–2 supervisors per exercise) all participants throughout each training session to reduce deviations from the study protocol and to ensure participant safety. Participants performed RT using a combination of free weights and machines. The RT protocol consisted of a whole-body program with eight exercises performed in the following order: chest press, horizontal leg press, seated row, leg extension, preacher curl, leg curl, triceps pushdown, and seated calf raise. Participants performed either three sets of 12/10/8 RM (NPR) or 15/10/5 RM (WPR) with incrementally higher loads for each set (crescent pyramid) [21,22,23]. The supervisors adjusted the 12/10/8RM and 15/10/5RM loads of each exercise according to the participant’s ability and improvements in exercise capacity throughout the study to ensure that participants used as much resistance as possible while maintaining proper technique. The load was increased 2–5% for the exercises of the upper limbs and 5–10% for the lower limbs. The participants were instructed to inhale during the eccentric muscle action and exhale during the concentric muscle action, while maintaining the velocity of movement at a ratio of 1:2 s for the concentric and eccentric phases, respectively. The rest interval ranged between 60–120 s for sets and 120–180 s for exercises. Participants were instructed not to perform any other type of physical exercise throughout the study period. The loads and the number of repetitions performed during each set of the eight exercises were individually recorded for each training session. The volume for each set of all exercises was calculated by multiplying the load by the number of repetitions. The volume of each exercise per session was calculated as the sum of the volume of all three sets for each exercise. The total volume load per session was calculated as the sum of all eight exercises. The weekly volume-load (WVL) was calculated by summing the three training sessions performed in one week. Increases in WVL throughout the RT program were calculated as the WVL of the eighth week minus the WVL of the first week.

### 2.6. Dietary Intake

Food intake was assessed by the 24-h dietary recall method applied on two non-consecutive days of the week, with the aid of a photographic record taken during an interview. Dietary intake was monitored in the first and last two weeks of the intervention period. The homemade measurements of the nutritional values of food were converted into grams and milliliters by the online software Virtual Nutri Plus (Keeple^®^, Rio de Janeiro, RJ, Brazil) for diet analysis. Some foods were not found in the program database and, therefore, these items were added from food tables.

### 2.7. Statistical Analyses

The Shapiro–Wilk test was used to analyze the distribution of data. A paired-samples t-test was performed to compare the total training volume of NPR and WPR groups. Data from all randomized participants have used an intention-to-treat analysis. Baseline data were repeated on post-intervention on dropouts. Generalized estimated equations (GEE) analyses were applied to investigate the effects of intervention over time within and between groups. Bonferroni post hoc test was adopted when significant effects on group, time, or interaction were confirmed. Effect size (ES) was calculated as pre-training mean minus post-training mean divided by the pooled pre-training standard deviation [26]. An ES of 0.00–0.19 was considered as trivial, 0.20–0.49 was considered small, 0.50–0.79 was considered moderate, and ≥0.80 was considered large [26]. The z-score of the percentage changes (from pre- to post-training) of the raw data for each parameter was calculated. A composite z-score derived from the average of the components was then calculated as the following formula: (TBF z-score + GLU z-score + TC z-score + TG z-score − HDL-C z-score + LDL-C z-score + CRP z-score)/7. For all statistical analyses, statistical significance was established at *p* < 0.05. The data were stored and analyzed using IBM SPSS Statistics, v. 22.0 (IBM Corp., Armonk, NY, USA).

## 3. Results

Fifty-five participants completed the intervention (CON = 18, NPR = 19, and WPR = 18) and four dropouts were registered, due to personal reasons (CON = 1) or training compliance lower than 85% (NPR = 1, WPR = 2). Baseline data were repeated in post-intervention in these cases. The total training volume was higher (*p* < 0.001) in WPR than in NPR (13,728.5 ± 950.9 kg vs. 12,534.3 ± 926.1 kg, respectively), as well as there were the greater progression in training loads and muscular strength gains favoring WPR [22]. There were no significant differences (*p* > 0.05) in macronutrients and daily relative energy within and between groups. Average intake was similar between them for carbohydrate (CON = 3.4 ± 1.2; NPR = 3.0 ± 1.0; WPR = 3.1 ± 1.0 g.kg.d^−1^), protein (CON = 1.0 ± 0.3; NPR = 1.0 ± 1.5; WPR = 1.0 ± 0.2 g.kg.d^−1^), lipid (CON = 0.7 ± 0.2; NPR = 0.7 ± 0.3; WPR = 0.7 ± 0.2 g.kg.d^−1^), and total energy (CON = 24.8 ± 7.4; NPR = 21.2 ± 7.8; WPR = 22.0 ± 5.6 kcal.kg.d^−1^). Table 1 presents the results on body fat at pre- and post-intervention according to groups. There was a significant interaction group by time in android body fat (*p* < 0.05) with increments in the CON group and a similar reduction for NPR and WPR. Compared to CON group, the ES of pre-to-post training changes (i.e., ES of training group minus ES of CON) were of trivial magnitude for total body fat (ES: NPR = −0.14; WPR = −0.16), small for android body fat (ES: NPR = −0.44; WPR = −0.53) and trivial for gynoid body fat (ES: NPR = −0.11; WPR = −0.12).

**Figure 1:**
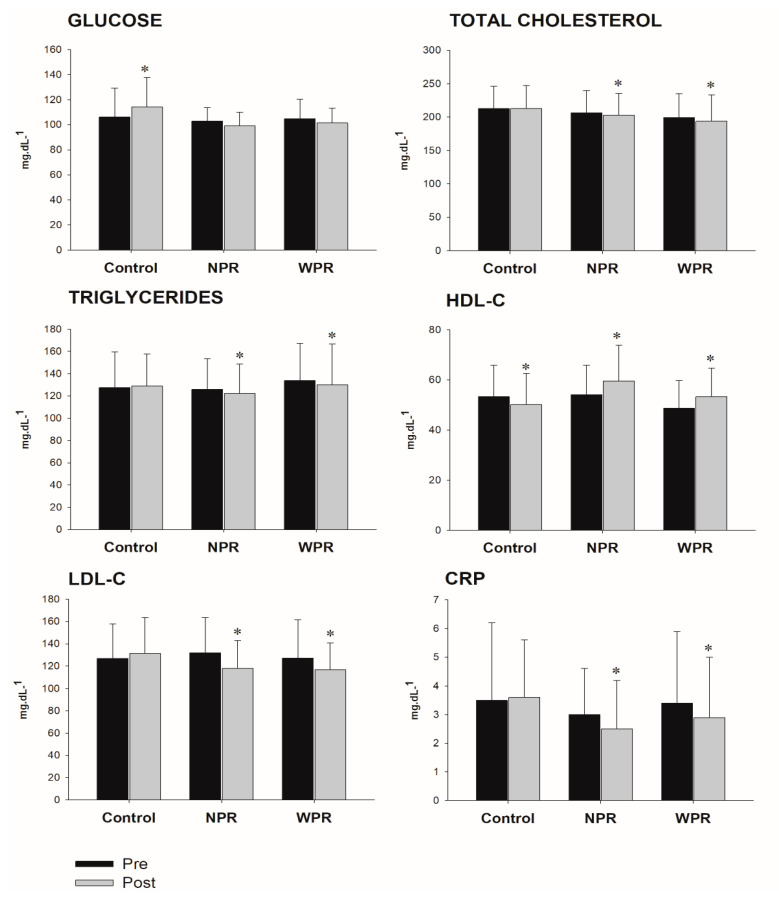
Blood markers at pre- and post-intervention according to groups. NPR = narrow repetition-zone pyramid training; WPR = wide repetition-zone pyramid training; HDL-C = high-density lipoprotein; LDL-C = low-density lipoprotein; CRP = high-sensitivity C-reactive protein. * *p* < 0.05 vs. pre.

**Figure 2:**
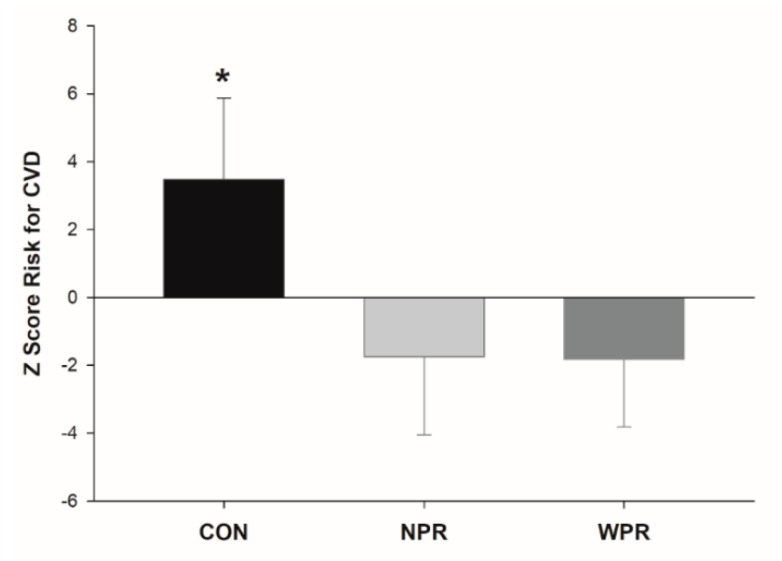
Composite Z-score of the percentage changes from pre- to post-training of the cardiovascular disease (CVD) risk according to groups in older women. CON = control group; NPR = narrow repetition-zone pyramid training; WPR = wide repetition-zone pyramid training; * *p* < 0.05 vs. NPR and WPR.

The effects on metabolic biomarker parameters are shown in Figure 1. Significant interaction group by time (*p* < 0.001) demonstrated benefits with RT for HDL-C (*p* < 0.001; CON = −6.1%; NPR = +10.3%; WPR = +9.4%), LDL-C (*p* < 0.001; CON = +3.9%; NPR = −10.9%; WPR = −8.3%), and CRP (*p* < 0.001; CON = +2.3%; NPR = −16.7%; WPR = −14.7%), however with similar magnitudes between NPR and WPR groups. Although the GLU have not been modified with RT, a significant group by time interaction effect was observed (*p* < 0.001), with an increase in the CON (+7.2%). For TC and TG, NPR and WPR groups had significant reductions pre-to-post training, while CON had no change, and significant group by time interaction effects were not observed (0.05 < *p* < 0.15). The ES of pre-to-post changes were of small-to-moderate magnitude for GLU (ES: NPR = −0.33; WPR = −0.22), TC (ES: NPR = −0.10; WPR = −0.15), TG (ES: NPR = −0.13; WPR = −0.11), HDL-C (ES: NPR = 0.47; WPR = 0.42), LDL-C (ES: NPR = −0.45; WPR = −0.30) and CRP (ES: NPR = −0.31; WPR = −0.20). The Z-score of CVD risk factors, created by the average of the intervention effects on the outcomes, indicate similar responses between NPR and WPR, differing from CON (Figure 2).

## 4. Discussion

The main finding of our study was that the crescent-pyramid RT system resulted in an attenuation of CVD risk factors following eight weeks of intervention in untrained older women. No difference between WPR and NPR was observed. Despite the higher volume–load performed by the WPR group, this did not influence the results, contradicting our initial hypothesis.

The mechanisms by which RT-induced body fat loss occurs, especially ectopic fat deposited in the android region, have not yet been fully elucidated [2,3,4,27]. However, it is possible that due to higher levels of estrogen and progesterone in older women, an increment in lipid oxidation rates occurs during exercise [6]. Moreover, in senescence, body fat becomes dysfunctional and is redistributed from subcutaneous to visceral and intra-abdominal deposits, beyond ectopic sites, including bone marrow, muscle, and liver [27]. Strategies to reduce regional body fat are then deemed necessary. Previous RT interventions with durations of 12 and 24 weeks showed reductions in android fat of −3% [28] and −12% [29] in untrained older women, indicating a benefit for the RT, and that training effects may be dose-response [24,30]. In parallel, we observed a reduction in android fat of 8% and 4% for WPR and NPR, respectively. However, this difference was not significant. Nonetheless, the CON group gained 11% fat. In this sense, the ES of the changes in body fat was of trivial to small magnitudes for training groups, compared to CON.

The increase in android fat deposits, especially visceral fat, enhances the risk of cardiovascular and metabolic diseases, by also inducing negative changes in metabolic blood profiles [2,3,4]. In our study, although no difference was found for GLU in the training groups, significant increases were revealed in the CON group (+8.0 mg.dL^−1^), indicating a clinical benefit for the RT on attenuating increases of this parameter over time, due to the lack of exercise practice. Although the glucose metabolism does not seem to be affected by the RT acutely [31], reductions induced by the continuous training was an expected response, considering the glycolytic demands for energy production associated with the exercise model [32]. Additionally, meliorations in glycemic and lipid profiles induced by RT have been related to body fat decreases [18].

The association between effects on body fat and lipid profile concerns the insulin-resistance condition and the increased circulating levels of free fatty acids and their impact on fat tissue [33]. In these circumstances, there are increases in the formation of large TG-rich LDL particles, which then decreases the expression of key enzymes related to fat loss in the plasma, such as the lipoprotein lipase [18,33]. In the same way, our results revealed significant reductions in CRP for both training groups, without difference between them. Indeed, RT seems to be a valid intervention strategy for improving inflammatory indicators such as the CRP [19,24,34,35]. Attenuating inflammation level is essential in the elderly because it is associated with numerous aging-related diseases, including hypertension, cardiovascular disease, diabetes mellitus, and kidney dysfunction [36].

Some issues of the current experiment are worth noting. The 8-week intervention period may not have been sufficient to differentiate the adaptive responses to the different repetition zones. Considering that some adaptations on the markers here explored are time-dependent [24], changes within and between groups would be more pronounced with longer training interventions. Additionally, although DXA is well established as a valid measure for determining body composition, subtle changes in body adiposity and changes in visceral fat would be detected with differents imaging equipment, such as computed tomography. Moreover, although we instructed women not to engage in any other physical exercise while participating in the study (of the three groups), no strict control was possible. No physical activity questionnaires were performed to guarantee such a point. On the other hand, monitoring habitual food consumption and the individual supervision of the RT sessions are strengths of the present work.

## 5. Conclusions

Our results indicate that narrow or wide repetitions zones of the pyramidal RT system similarly reduced the cardiovascular risk factors in older women. The use of the pyramid training system may be an interesting alternative to the traditional scheme for the prescription of RT to older women since it allows to train with a progressive overload for each set and gradually increase the muscle stimuli, which makes work more efficient and the training session more motivating and challenging.

## Figures and Tables

**Table 1:** Body fat at pre- and post-intervention according to groups.

Variables		Control (*n* = 19)	Narrow Repetition Zone (*n* = 20)	Wide Repetition Zone (*n* = 20)	Interaction *p*−Value
Total body fat (kg)	Pre	28.0 ± 10.5	26.8 ± 8.9	29.2 ± 7.9	0.14
	Post	28.8 ± 11.8	26.4 ± 10.0 *	28.6 ± 7.8 *	
	∆%	+2.8	−1.5	−2.1	
Android body fat (kg)	Pre	2.5 ± 1.2	2.6 ± 0.8	2.8 ± 1.0	<0.01
	Post	2.8 ± 1.0 *	2.5 ± 0.9 *	2.6 ± 1.2 *	
	∆%	+10.8	−4.3	−8.1	
Gynoid body fat (kg)	Pre	4.9 ± 1.5	5.1 ± 1.6	5.1 ± 1.3	0.63
	Post	4.9 ± 1.7	5.0 ± 1.7 *	5.0 ± 1.2 *	
	∆%	+0.4	−2.0	−2.0	

Note. Data are expressed as mean ± standard deviation. * *p* < 0.05 vs. pre.
